# High carbohydrate diet programs metabolic enzyme gene expression modification in F2 generation wistar rat males

**DOI:** 10.1016/j.crphys.2025.100154

**Published:** 2025-06-18

**Authors:** Joseph Chimezie, Worship Odosa Agbonifo, Hope Oluwabukola Francis, Mercy Oluwaseun Awoleye, Temitope Gabriel Adedeji

**Affiliations:** a Epigenetics and Molecular Biology Laboratory, Federal University of Technology, Akure, Nigeria; b Department of Physiology, School of Basic Medical Sciences, Federal University of Technology, Akure, Nigeria

**Keywords:** Filial generation, Gene expression, High carbohydrate diet, Metabolic enzymes

## Abstract

Diets high in carbohydrates (HCD) negatively impact transgenerational metabolic health and phenotype, factors directly influenced by gene expression. This study investigates the effects of HCD feeding on gene expression of key enzymes of important metabolic pathways in the Parent (F0), first (F1) and second (F2) filial generations. Each generation consisted of a control and HCD group of male and female counterparts in the F0 and F1 generations. Female rat cohorts (F0) fed a control or high-carbohydrate diet were mated at pro-oestrous period with males fed with similar diets at a ratio of 1:1 overnight. The offspring of the F1 generation exposed to the same diet were mated (1:1) to produce the F2 generation fed on a control diet. Male animals in each generation were analysed for metabolic changes, gene expression, and phenotypic outcomes. The results indicate that HCD caused significant increases (P < 0.05) in body weight in both the F1 and F2 generations, fasting blood glucose in the F2 generation, as well as serum insulin and HOMA-IR in the F1 and F2 generations. The F0 and F1 HCD-fed rats demonstrated a significant increase (P < 0.05) in the expression of genes involved in glycolysis and glycogen synthesis, along with a significant decrease (P < 0.05) in the expression of genes for gluconeogenic enzymes. Additionally, there was an increase (P < 0.05) in the expression of genes associated with fatty acid biosynthesis and a decrease (P < 0.05) in β-oxidation gene expression, a pattern similarly observed in control-fed F2 male rats. These findings suggest that a parental diet high in carbohydrates can induce modifications in the gene expression of metabolic rate-limiting enzymes in F2 offspring, regardless of their diet. However, this study did not assess the epigenetic modifications potentially responsible for the observed transgenerational effects. Future research could investigate epigenetic changes such as DNA methylation and histone modifications, and also assess these effects in female animals.

## Highlights

• F2 exhibited weight gain and glucose intolerance, similar to HCD-fed F0 and F1 ancestors.
• HCD reduced fatty acid oxidation by downregulating key enzymes ACOX-1 and CPT-1.
• Increased glucose and insulin from HCD stimulated lipogenic gene expression (FAS and ACC) across generations.
• HCD-fed generations showed increased glycogen synthase expression, inherited by F2 generation.
• HCD reduced PEPCK expression, affecting future generations.

## 1 Introduction

Nutrients provide essential raw materials and building blocks for numerous metabolic processes in every body cell (Chen et al., 2018). Prolonged disturbances in nutrient metabolism and energy balance can lead to cellular stress, tissue damage, and overall metabolic dysfunction (Chen et al., 2018). Lifestyle and dietary choices, such as consuming processed foods and high-fat, high-sugar, or high-protein meals, directly affect nutrient availability, metabolism, and homeostasis, impacting health and contributing to the rise in metabolic disorders like obesity and metabolic syndrome (Chen et al., 2018; Angelico et al., 2023). Metabolic alteration during early life, evident in a poor maternal diet associated with high-energy dietary intake, suggests mechanisms of altered intrauterine environment, providing an understanding of the mechanisms of transgenerational transmission of metabolic disorders into future generations from parental (F0) to second filial generation (F2) (Aiken et al., 2016; Vickers, 2014). These mechanisms of transgenerational effects remain poorly understood, although evidence of alteration in endocrine function regulation, energy homeostasis and epigenetic modification has been reported (Thorsell and Nätt, 2016; Reynolds et al.; Guo et al., 2020). Poor nutritional health in parents has been reported to confer negative consequences beyond immediate offspring, persisting across several generations, including the F2 and F3, despite no direct exposure to the dietary intervention (Thorsell and Nätt, 2016).

Diet rich in carbohydrate-related metabolic syndrome has been linked with impaired lipid and glucose metabolism, attributed to hyperinsulinemia, accumulation of glycogen, high pyruvate, lactic acid, triglyceride (TG) and free fatty acids (NEFA) levels in plasma, low high-density lipoprotein (Zhao et al., 2022; Parks and Hellerstein, 2000; Adedeji et al., 2019). Carbohydrates play essential roles in energy provision, structural support, and cellular signaling, making them vital for normal fetal development during pregnancy (Okburan et al.; Xue et al., 2024a). However, high carbohydrate intake during pregnancy has been closely linked to an increased risk of metabolic dysfunction, adversely impacting offspring development (Xue et al., 2024b). Nutrient homeostasis involves complex regulation, balancing metabolic and hormonal adjustments through diet-induced gene expression changes (Tiffon, 2018). High-carbohydrate diets (HCDs) during pregnancy influence various metabolic pathways, such as increasing plasma levels of pyruvate kinase (PK), triglycerides (TG), and free fatty acids (FFA) (Zhao et al., 2022; Agius, 2016). HCDs also contribute to elevated liver glycogen levels and upregulate the activity of genes linked to lipid synthesis, such as phosphoenolpyruvate carboxykinase (PEPCK), fatty acid synthase (FAS), acetyl-CoA carboxylase (ACC), and peroxisome proliferator-activated receptor gamma (PPAR-γ) (Zhao et al., 2022; Yang et al., 2023; Li et al., 2015). HCD also reportedly enhances inflammatory and apoptosis gene expression in fish intestines (Zhao et al., 2021, 2022). Furthermore, HCDs can lead to DNA methylation changes in genes related to lipid metabolism, such as sterol regulatory element-binding protein 1 (SREBP1), PPAR-α, and peroxisome proliferator-activated receptor coactivator 1-alpha (PCG1-α), potentially altering gene expression profiles (Cai et al., 2020). These diets have also been implicated in insulin resistance and obesity development in animal models, correlating with increased gene expression associated with glycolysis and gluconeogenesis (Raychaudhuri et al., 2014; Agius, 2013; Lu et al., 2022).

Changes in metabolic gene expression are closely linked to alterations in phenotype, as they can modify fundamental biochemical and physiological processes that determine an organism's observable traits (Dai et al., 2020). When specific genes involved in metabolism are upregulated or downregulated, these shifts can lead to differences in how cells process energy, store fat, or manage insulin sensitivity, directly influencing traits like body weight, fat distribution, and muscle mass. For instance, upregulation of lipid synthesis genes such as fatty acid synthase (FAS) or acetyl-CoA carboxylase (ACC) often results in increased fat storage, contributing to an obese phenotype (Badmus et al., 2022; Hodson and Gunn, 2019; Hammad and Jones, 2017). Similarly, changes in the expression of genes regulating glucose metabolism, such as phosphoenolpyruvate carboxykinase (PEPCK), can affect blood sugar levels and insulin response, potentially leading to phenotypic manifestations of metabolic disorders like type 2 diabetes (Hodson and Gunn, 2019; Hammad and Jones, 2017). Epigenetic modifications, such as DNA methylation in metabolic genes (e.g., PPAR-α or SREBP1), further reinforce these phenotypic shifts by establishing a “memory” of these gene expression changes, making the metabolic phenotype more stable and sometimes heritable across generations (Pant et al., 2020). Thus, by altering gene expression in metabolic pathways, environmental and nutritional factors can create sustained changes in phenotype, influencing health outcomes across the lifespan (Tiffon, 2018).

Maternal nutrition plays a critical role in shaping birth outcomes, with dietary choices and socioeconomic factors often contributing to inadequate nutrient intake that adversely impacts fetal development (Xue et al., 2024b; Abu-Saad and Fraser, 2010). Research indicates that epigenetic plasticity, our ability to modify gene expression based on environmental inputs, enables the fetus to adapt to changes in nutrient availability, which significantly affects birth characteristics linked to nutrition-dependent epigenetic processes (Kanherkar et al., 2014; Geraghty et al., 2015). Suboptimal maternal nutrition triggers metabolic and epigenetic changes in the developing fetus, leaving lasting marks on the progeny's epigenome (Geraghty et al., 2015; Abraham et al., 2023; Jiao et al., 2024). These changes manifest through various modifications, including shifts in gene expression, alterations in metabolic enzymes and signaling pathways, and a wide array of DNA modifications, such as methylation. Histone post-translational modifications also occur, encompassing processes like acetylation, phosphorylation, hydroxybutyrylation, lactylation, ubiquitination, succinylation, and acylation, each contributing uniquely to metabolic programming in the fetal epigenome (Jiao et al., 2024; Sun et al., 2022; Huo et al., 2021).

The process of metabolic programming, initiated by maternal exposure to environmental stress, highlights the profound influence of prenatal factors on long-term health trajectories (Vaiserman, 2015). These adaptations, primarily epigenetic, occur as a response to nutrient stress, which may predispose offspring to metabolic disorders by setting regulatory pathways during early development (Geraghty et al., 2015; Maugeri et al.; Amorin et al., 2023). Specifically, metabolic fetal programming has been recognized as a potent contributor to the development of metabolic syndrome in adulthood (Zhu et al., 2019). Moreover, parental exposure to specific macronutrients has been shown to impact offspring metabolism significantly, even when balanced nutrition interventions are introduced, due to enduring epigenetic changes (Dimofski et al., 2021). Beyond epigenetic modifications, additional mechanisms such as hormonal imbalances and oxidative stress can alter developmental trajectories and metabolic function in offspring exposed to abnormal macronutrient environments (Dupont et al., 2019).

The rising prevalence of metabolic disorders, such as obesity and type 2 diabetes, has prompted investigation into the transgenerational impacts of dietary habits, particularly high-carbohydrate diets (HCDs). Recent studies suggest that dietary intake by parental generations may alter gene expression not only in the immediate offspring (F1) but also in subsequent generations (F2). However, the specific mechanisms and extent to which diet-induced gene expression changes persist in F2 generation Wistar rats remain poorly understood. This study hypothesizes that multigenerational exposure (F0 and F1) to a HCD may lead to persistent alterations in phenotype and the expression of genes related to metabolic enzymes in the F2 progeny. To evaluate this, blood glucose levels, serum insulin concentrations, and the activity of key hepatic metabolic enzymes involved in glucose and lipid metabolism will be assessed. Understanding these transgenerational effects could provide insight into how dietary patterns contribute to inherited susceptibility to metabolic diseases, highlighting potential targets for early intervention in at-risk lineages.

## 2 Methods

### 2.1 Animals and diets

All experimental procedures were carried out in compliance with the Guide for the Care and Use of Laboratory Animals (N.R.C.U.C.f.t.U.o.t.G.f.t.C.a.U.o.L. Animals). Male and female Wistar rats for the F0 generation were purchased after weaning (PND 21) from the Federal University of Technology Animal Breeding Facility Akure, Nigeria. The animals were housed in well-aerated experimental animal cages, kept on a 12-h light/dark cycle, maintained at room temperature (22 ± 2 °C) with a relative humidity of 50–60 % in the animal house of the Department of Physiology Federal University of Technology Akure, Nigeria. They were fed on standard rat chow (Ladokun feeds, Nigeria Limited) and had free access to clean drinking water during a 7-day acclimating period prior to the commencement of the dietary grouping and feeding. All females were nulliparous, and the males used were proven breeders (fertility confirmed by the isolated mating technique).

### 2.2 Animal feed

Composition of each diet was determined and is as follows.

• Group 1 (Control): Normal rats' chow (26.5 % protein, 40 % carbohydrates, 29 % fat, and 4.5 % crude fibre) amounting to 3.2kCal/g
• Group 2: High Carbohydrate Diet (HCD) (20 % protein, 58.5 % carbohydrates, 17 % fat, and 4.5 % crude fibre) amounting to 4.4kCal/g

The diets were designed to contain at least 20 % total protein to provide essential amino acids, in line with the recommendations of the National Research Council (US) Subcommittee on the Tenth Edition of the Recommended Dietary Allowances (N.R.C.U.S.o.t.T.E.o.t.R.D. Allowances). Composition of the diets are shown in Table 1. Animals were housed individually in metabolic cages. A pre-weighed amount of feed was provided to each cage, and the remaining feed was collected and weighed after 24 h. The difference between the initial and remaining feed weight was recorded as the daily food intake. This process was repeated daily throughout the feeding period to monitor and compare food consumption trends across the dietary groups.

**Table 1:** Composition of standard diet and HCD for animal feeding.

S/N	Component	Standard Chow (kg)	HCD (kg)
1	Soya	10	6
2	Palm Kernel Cake	4	4
3	Wheat offal	10	10
4	Premix	0.1	0.1
5	Groundnut Cake	10	6
6	Maize	10	18
7	Lysine	0.1	0.1
8	Methionine	0.1	0.1
9	Fish meal	4	4
10	Bone Meal	2	2
11	Salt	0.1	0.1
12	Lard	0	0
	Total	50.4	50.4

## 3 Experimental protocol

### 3.1 Animal grouping and diet administration

Twenty (20) Wistar rats (10 males and 10 females, PND 21) were used for the study as the first filial (F0) generation. Based on preliminary data and previous studies on metabolic parameters in rats, we anticipated a moderate to large effect size (Cohen's d ≈ 0.8). Using an alpha of 0.05 and a power of 0.8, power analysis (conducted using G∗Power) indicated that a minimum of 8–10 animals per group would be sufficient to detect meaningful differences. Therefore, our use of 10 animals per group is statistically justified and aligns with ethical considerations to minimize animal use while maintaining adequate statistical power for transgenerational analysis. The animals were fed on experimental and control diets for twenty (F0 and F1) or nine (F2) weeks as summarised in Table 1.

### 3.2 Mating procedure

During the feeding period, the female rat's oestrous cycle was assessed daily as described by Marcondes et al. (2002). They were further isolated during the pro-oestrous period and mated with males fed with similar diets at a ratio of 1:1 overnight (Reddy et al., 2022). Meanwhile, mating was confirmed as indicated by the presence of sperm in the vagina, or a copulatory plug the next morning and marked as the first day of gestation. Of the progeny obtained from the female and male control and experimental animals second filial generation (F1) was obtained and further isolated as F1 sibling pairs (10 males and 10 females per group) were randomly selected. The F1 sibling pairs were also fed on the control and HCD diet for twenty (20) weeks after a three-week weaning period. For the F2 generation, we randomly selected F1 males from the control and experimental animal groups, randomly paired with selected F1 females of 12 weeks old (1:1) for 2 weeks. Following daily vaginal smear checks, the female F1 cohorts were confirmed positive on the first day of gestation. The animals were maintained on the diets throughout gestation, till childbirth on positive vaginal smear confirmation. The male offspring F2 were fed on the control diet for 9 weeks after weaning.

### 3.3 Tissue collection

**Fig. 1:**
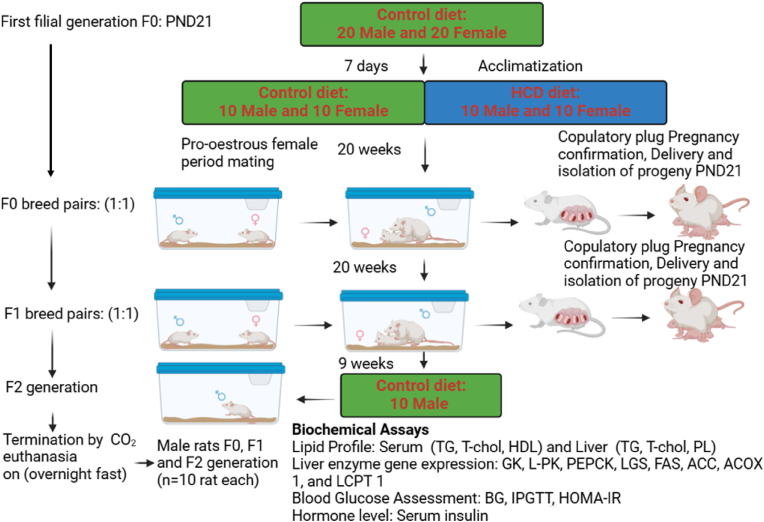
**Experimental plan of control and HCD-fed rat cohort across different** generations**.** F0 generation (n = 10 male and 10 female animals fed on control and HCD-fed diets), F1 generation (n = 10 male and 10 female progeny fed on the same diets as F0) and F2 generation progeny of F1 (n = 10 male animals fed only the control diet).

After the experimental treatment period, 10 male animals in both generations (F0, F1, F2) each were fasted overnight, anaesthesized with a 100 mg/kg ketamine and 10 mg/kg xylazine cocktail administered intraperitoneally, then euthanized by CO_2_ inhalation intraperitoneallyand euthanized (Fig. 1). Liver and serum samples were taken, immediately and frozen for biochemical analysis, since a previous study and a preliminary pilot study we conducted had shown that female animals did not respond significantly to dietary changes within the experimental period (Kulhanek et al., 2020; Adedeji et al., 2017).

### 3.4 Blood glucose assessment

Fasting blood glucose was measured in 6-h fasted animals by pricking the lateral tail vein with a sterile lancet. Rats were fasted beginning at 8:00 a.m. and blood glucose measurements were performed at 2:00 p.m., thereby ensuring a consistent 6-h fasting period for all experimental animals The obtained blood drop was measured for glucose concentration using an AlphaTRAK2 Blood Glucose Monitor and standard strips (ADW Diabetes, USA).

### 3.5 Intraperitoneal glucose tolerance test

Intraperitoneal glucose tolerance test (IPGTT) was performed on male rats of F0, F1 and F2 generation to determine the glucose metabolism and insulin response in each rat. Concisely, following a 6-h fast with free access to water of male experimental rats the baseline blood glucose levels were obtained from tail blood samples via tail incision using glucose oxidase method (Small et al., 2022; Bruce et al., 2021; Watada et al., 2014). Glucose load solution (2 g/kg) was administered to rats intraperitoneally and blood glucose levels were recorded at 0, 20, 40, 60, and 120 min to determine glucose absorption and insulin consumption activity from the area under curve (AUC).

### 3.6 Biochemical assays

Blood was collected via cardiac puncture for the male cohorts of different filial generations (F0, F1 and F2) after CO_2_ euthanasia. The blood obtained was allowed to stand for 2 h and centrifuged at 8000 g for 10 min at room temperature. The supernatant was collected, and the centrifuge step was repeated.

### 3.7 Determination of the lipid profile

Collected serum was assayed for triglyceride (REF; 51215002), total cholesterol (REF; 51403002), and high-density lipoprotein cholesterol (REF; 51010001) using enzymatic quantitation kits obtained from AGAPPE diagnostics, Switzerland. Extraction and purification of lipids from the liver were done according to the method of Folch (Folch J and Stanley Sloane, 1957). This was followed by the determination of triglyceride, total cholesterol, and phospholipid concentration in the liver according to the methods of Fletcher (1968), and Sperry and Webb (1950), and Bartlett (1959). Briefly, 2 μL of liver tissue, serum and standard were mixed with 200 μL of working reagent (triglyceride, cholesterol, phospholipids), incubated at 37^o^C for 5 min, and absorbance read at 500 nm using a microplate reader to determine the triglyceride (mmol/l), cholesterol level (mg/dl). 300 μL of serum and 300 μL of precipitating reagent were mixed, incubated for 10 min at room temperature and centrifuged at 4000 rpm for 10 min to obtain a clear supernatant containing HDL. Then, 5 μL of supernatant or HDL standard was mixed with 200 μL of working reagent in a 96-well plate, incubated at 37 °C for 5 min and read at 500 nm to determine HDL (mg/dL).

### 3.8 Determination of hormone level

Using Enzyme-Linked Immunosorbent Assay (ELISA) kits provided by Bioassay Laboratory Technology (Wuhan, China), serum insulin (Catalogue; ER1113) levels were determined. Briefly, 100 μL of serum sample and standard were pipetted into pre-coated 96-well plates and incubated for 90 min at 37 °C and decanted. Immediately, 100 μL of biotinylated detection antibody was added to each well, incubated for 60 min at 37 °C, decanted and washed 3 times, then 100 μL of avidin-Horseradish peroxidase (HRP) conjugate was added to each well, incubated for 30 min at 37 °C and washed 5 times. 90 μL of (3,3′,5,5′-Tetramethylbenzidine (TMB) substrate solution was added into each well, incubated for 15 min at 37 °C in the dark, and then 50 μL of stop solution. The absorbances were read at 450 nm using a microplate reader. The concentration of insulin in the serum sample was obtained (pg/mL) by comparing their absorbance value to the standard curve.

### 3.9 Determination of insulin resistance

Using the obtained glucose level (mg/dL) and serum insulin (μU/mL), the homeostasis model assessment of insulin resistance (HOMA-IR), was determined by calculating the insulin resistance index (HOMA-IR) = [fasting glucose (mg/dL) × fasting INS(pg/mL)]/405 (Breneman and Tucker, 2013; Biernacka-Bartnik et al., 2023).

### 3.10 RT-qPCR for gene expression of enzymes of metabolism

Liver enzyme gene expression was measured by performing Real Time PCR on the ABI 7300 (Applied Biosystems) using Power Sybr Green PCR mix (Applied Biosystems). As listed in Table 2, the primer sequences of genes used in this study include; A) primer for glycolysis: glucokinase (GK); liver pyruvate kinase (L-PK), (B) gluconeogenesis: phosphenolpyruvate carboxykinase (PEPCK), (C) glycogen synthesis: glycogen synthase (liver isoform) (GS); (E) lipid synthesis: fatty acid synthase (FAS); acetyl- CoA carboxylase (ACC), (F) lipolysis: acyl-CoA oxidase (ACOX 1); carnitine acyltransferase 1 (liver isoform) (CPT 1). Briefly, 10 ng (5 ml) of cDNA vortexed with the addition of 15 ml of the reagent containing RNAase free water, PCR mix, forward and reverse primers were used. Reactions were performed as follows: denaturation for 10 min at 95 μC, 40- times at 95 μC for 15 s followed by 1 min at 60 μC (amplification). 18 S rRNA was used as the standard. The threshold (CT) was set with the constant value for all of the genes and samples to quantify the mRNA concentration. The negative controls were used to notify the contamination (control without RT or RNA). The efficiency was estimated using series of 5-fold dilution of the sample and checked for each run.

**Table 2:** Primer sequences for enzymes of metabolism.

GENE	SEQUENCE
**ACC**	Up: 5′-CAACGCCTTCACACCACCTT-3′
	Down: 5′-AGCCCATTACTTCATCAAAGATCCT-3′
**ACOX-1**	Up: 5′-AAGAAATCCCCACTGAACAAAACA-3′
	Down: 5′-CCCAGGGAAACTTCAAAGCTT-3′
**CPT1**	Up: 5′-ATATCAAGGACAGCAGGCACAT-3′
	Down: 59-CTCAGCAGCCTCCCATGCT-3′
**FAS**	Up: 5′-TGCTCCCAGCTGCAG-3′
	Down: 5′-GCCCGGTAGCTCTGGGTGTA-3′
**GK**	Up: 5′-TTGAGACCCGTTTCGTGTCA-3′
	Down: 5′-AGGGTCGAAGCCCCAGAGT-3′
**GS**	Up: 5′-GACACTGAGCAGGGCTTTTCC-3′
	Down: 5′-GAGGAGGGCCTGGGATACTT-3′
**LPK**	Up: 5′-TGATGATTGGACGCTGCAA-3′
	Down: 5′-GAGTTGGTCGAGCCTTAGTGATC-3′
**PEPCK**	Up: 5′-GAAAGTTGAATGTGTGGGTGAT-3′
	Down: 5′-TTCTGGGTTGATGGCCCTTA-3′

Gene expression was calculated as: 2^−ΔC^, where ΔC = C_T_ Gene - C_T_ 18 S.

### 3.11 Statistical analysis

Data are expressed as mean ± SEM. The significance of differences among dietary groups within each generation was assessed using Student's t-test and two-way analysis of variance (ANOVA). Post-hoc comparisons between group means were performed using the Tukey-Kramer multiple comparison test, selected for its ability to control type I error when comparing multiple group means. Specifically, we selected this test because it is appropriate for pairwise comparisons among group means when sample sizes are unequal, and controls the family-wise error rate, ensuring the reliability of multiple comparisons. P < 0.05 was regarded as statistically significant.

## 4 Results

### 4.1 Body weight

Body weight of F1 generation rats fed on HCD was significantly higher (p < 0.05) when individually compared with control (Table 3). Similarly, in the F2 generation, HCD showed significantly higher body weights when compared with the control, even though the animals only had the control diet (Table 3).

**Table 3:** Effect of diets on body weight (g) in Wistar rats across generations.

	F0 Generation	F1 Generation	F2 Generation
Control	276 ± 5.8	282 ± 4.4	221 ± 7.2
HCD	282 ± 6.3	288 ± 3.8∗	236 ± 5.4∗

Values are mean ± SEM for ten male animals per dietary group. ∗P < 0.05 vs control, significant. HCD: high carbohydrate diet.

### 4.2 Average feed consumption of offspring

In F1 and F2 generations, there was no significant difference in feed consumption between the groups, and animals ate almost the same quantities of the feed irrespective of the diet. However, because of the difference in calorie content, this amounted to an increase in calorie intake in F1 generation offspring fed on HCD. Since F2 rats were fed the same control diet, calorie intake stayed same in HCD-fed rats and controls. (Table 4).

**Table 4:** Average feed consumption of offspring.

F1 Generation		
Diet	Feed Consumption (g)	Calorie intake kcal/g/day
Control	18.5 ± 0.5	59.2 ± 3.4
HCD	18.3 ± 0.6	79.8 ± 4.2∗

F2 Generation		
Diet (fed Control)	Feed Consumption (g)	Calorie intake kcal/g/day
Control	18.7 ± 0.6	59.8 ± 3.2
HCD	18.1 ± 0.7	57.9 ± 5.2∗

Values are mean ± SEM for ten male animals per dietary group: ∗P < 0.05 vs control, significant. HCD: high carbohydrate diet.

### 4.3 Effect of high carbohydrate diet on glucose metabolism and insulin resistance in wistar rats across generations

**Fig. 2:**
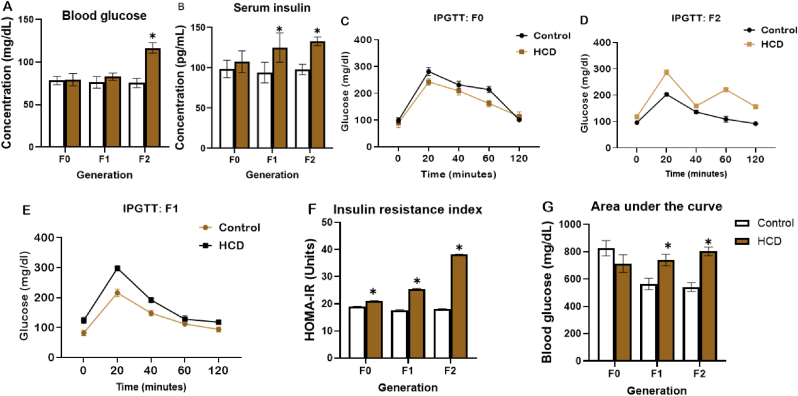
Effect of high carbohydrate diet on blood glucose, serum insulin, glucose clearance and HOMA-IR in Wistar rats across generations. (A) Blood glucose, (B) Serum insulin (C–E) Intraperitoneal glucose tolerance test (F) HOMA-IR (G) Area under the curve. Values are mean ± SEM for ten male animals per dietary group: ∗P < 0.05 = significant in comparison with control. F0, F1 and F2 (first, second and third generations respectively). HCD: high carbohydrate diet, IPGTT: Intraperitoneal glucose tolerance test, HOMA-IR: Homeostatic assessment of insulin resistance.

**Fig. 3:**
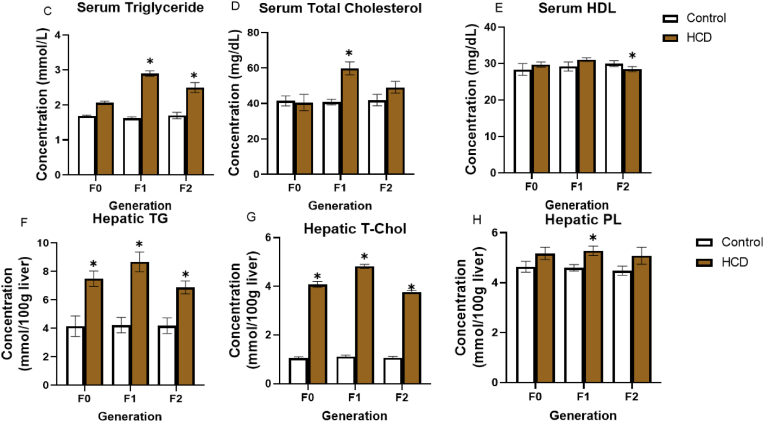
Effect of high carbohydrate diet on blood glucose, serum insulin, glucose clearance and HOMA-IR in Wistar rats across generations. (A) Blood glucose, (B) Serum insulin (C–E) Intraperitoneal glucose tolerance test (F) HOMA-IR (G) Area under the curve. Values are mean ± SEM for ten male animals per dietary group: ∗P < 0.05 = significant in comparison with control. F0, F1 and F2 (first, second and third generations respectively). HCD: high carbohydrate diet, IPGTT: Intraperitoneal glucose tolerance test, HOMA-IR: Homeostatic assessment of insulin resistance, T-Chol; Total cholesterol, PL; phospholipids, TG; triglyceride, HDL; high density lipoprotein.

Fasting blood glucose level (Fig. 2A) was significantly increased in F2 generation fed HCD at the end of the feeding period when compared with the control while serum insulin concentration (Fig. 2B) was increased in HCD-fed rats of the F1 and F2 generations when individually compared with their respective controls. As depicted in Fig. 2C–E, the intraperitoneal glucose tolerance test indicated decrease blood glucose at 0–120 min in F0 and F1 HCD fed cohort when compared to their control counterparts. On the other hand, F2 generation not fed on the experimental diet showed increased blood glucose at 0–120 relative to control. Area under the curve showed a significant increase in blood glucose in F1 and F2 generation when compared with their controls. As illustrated in (Fig. 2F and G), the index of insulin resistance (HOMA-IR) showed a significant increase across all generations relative to their controls (see Fig. 3).

### 4.4 Effect of high carbohydrate diet on serum and liver homogenate lipid profile concentration in wistar rats across generations

Serum triglyceride levels were increased in HCD groups in the F1 and F2 generations when compared to their control. Serum total cholesterol levels were also significantly elevated in HCD fed rats of the F1 generation in comparison with the control. However, HCD diet resulted in a significant decrease serum HDL concentration in the F2 generation.

Liver homogenate triglyceride concentration was increased in all generations on the HCD when individually compared against their respective generation controls. Total cholesterol level was increased in the liver homogenate of HCD-fed rats in F0, F1, and F2 generations when individually compared with their generation control. The concentration of liver homogenate phospholipids was increased in the HCD group of the F1 generation.

### 4.5 Effect of different diets on expression of glucokinase gene

**Fig. 4:**
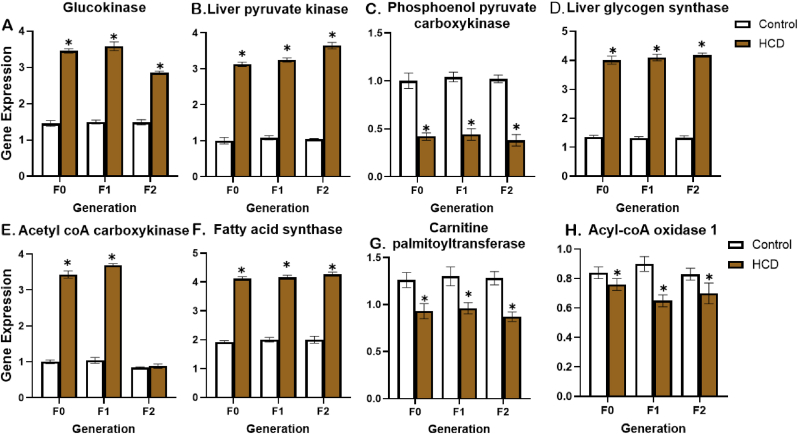
Effect of high carbohydrate diet on liver enzyme gene expression of F0, F1 and F2. generations. GK: glucokinase, LPK: pyruvate kinase, PEPCK: Phosphoenolpyruvate Carboxykinase, LGS: glycogen synthase, ACC: acetylcoA carboxykinase, FAS: fatty acid synthase, CPTK: carnitine palmitoyltransferase and ACOX-1: acylcoA oxidase. Values are mean ± SEM for ten male animals per dietary group; P < 0.05 ∗ = significant in comparison with control.

**Fig. 5:**
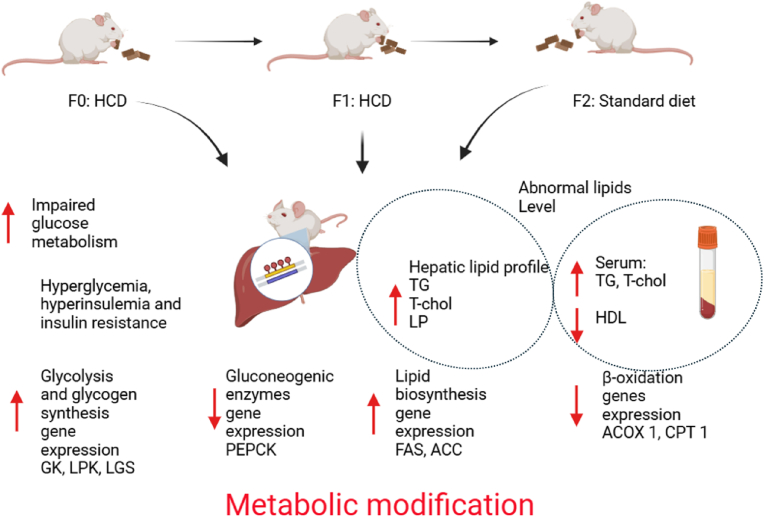
Schematic summary of metabolic and epigenetic modification of dietary consumption high in carbohydrate in F0, F1 and F2 generations. TG: triglyceride, T-chol: total cholesterol LP: lipoprotein, HDL: high density lipoprotein, GK: glucokinase, LPK: liver pyruvate kinase, LGS: liver glycogen synthase, PEPCK: phosphoenolpyruvate carboxykinase, ACC: acetyl-CoA carboxylase, FAS: fatty acid synthase, CPT1: carnitine palmitoyltransferase 1, ACOX1: acyl-CoA oxidase 1.

Results from Fig. 4A show that on individual comparison with their respective controls, the HCD group had a significant increase in glucokinase gene expression in F0, and F1 generations. Similarly, the HCD in the F2 generation displayed a significant increase in glucokinase gene expression (see Fig. 5).

Analysis of results from Fig. 4B, show that the HCD group had a significant elevation (P < 0.05) of liver pyruvate kinase expression in F0 and F1 generations. The offspring of HCD-fed animals (F2) also reflected a significant increase in pyruvate kinase gene expression. However, phosphoenolpyruvate carboxykinase levels showed a significant decrease in expression across all generations compared to their respective controls (Fig. 4C).

Glycogen synthase gene expression levels were significantly increased in HCD fed rats across all generations in comparison with their control (Fig. 4D).

Acetyl coA carboxykinase gene expression levels in F0 and F1 rat corhorts fed with HCD reflected a significant increase (P < 0.05) when compared with their controls (Fig. 4E). Similarly, fatty acid synthase expression levels were significantly increased in rats fed with HCD across all generations in comparison with their controls (Fig. 4F).

Additionally, Carnitine palmitoyltransferase-1 gene expression was significantly reduced (P < 0.05) in rats fed on the HCD across all generations compared to their controls (Fig. 4G). Acyl-CoA oxidase 1 expression was also significantly downregulated in the HCD-fed rats across all generations when compared with their respective controls (Fig. 4I).

## 5 Discussion

Multigenerational exposure to energy-dense diets, including those rich in carbohydrates, has been linked to significant alterations in metabolic phenotypes such as body weight and energy homeostasis (Adedeji et al., 2019). In this study, HCD feeding in the F1 generation resulted in increased body weight and caloric efficiency. Notably, this phenotype was also observed in the F2 progeny that were maintained on a standard control diet, despite no significant change in overall food intake. These findings suggest a transgenerational metabolic programming effect, in which exposure to HCD in the parental lineage predisposes offspring to enhanced energy storage or reduced energy expenditure. This phenomenon aligns with earlier research indicating that parental intake of energy-rich diets can lead to inheritable epigenetic modifications that affect energy balance regulation (Reynolds et al.; Milagro et al., 2013; Park et al., 2017). These modifications may influence hypothalamic signaling and appetite-regulating hormones. Such reprogramming may reflect an adaptation toward energy conservation but can become maladaptive in environments of nutrient excess (Bokor et al., 2024; Desai et al., 2015; Li et al., 2021). The increased body weight in F2 generation, even in the absence of dietary excess, support the idea that ancestral dietary intake can alter energy metabolism trajectories in descendants (Kaspar et al., 2020). Our design, with F0 and F1 generations fed a high-carbohydrate diet (HCD) and the F2 generation switched to a control diet, aimed to assess both parental and cumulative multigenerational dietary effects on metabolism. Prior studies show that prolonged exposure across generations amplifies metabolic programming, revealing changes not seen with single-generation exposure (Burdge et al., 2011; Li et al., 2012). Epigenetic changes, particularly during prenatal and early postnatal windows, may require multigenerational exposure to fully manifest (Waterland and Jirtle, 2003) (Waterland and Jirtle, 2003). By transitioning F2 to a control diet, we could distinguish inherited metabolic programming from direct dietary effects, aligning with findings where paternal diet altered offspring metabolism independent of their own diet (Carone et al., 2010; Ng et al., 2010).

Lipid profile analysis further revealed significant disruptions in F2 animals. The F2 progeny of HCD-fed parents showed signs of hyperlipidemia, hypertriglyceridemia, and reduced serum HDL, mirroring the dyslipidemia observed in both F0 and F1 generations (Adedeji et al., 2019). These findings suggest that ancestral dietary carbohydrate excess can induce heritable alterations in lipid metabolism. The reduction in HDL and increase in serum triglycerides and cholesterol are consistent with established markers of metabolic syndrome and may indicate a predisposition to hepatic steatosis, insulin resistance, and cardiovascular risk (Parhofer, 2015).

Simultaneously, F2 progeny also displayed impaired glucose regulation. Increased blood glucose and insulin levels, along with signs of insulin resistance and glucose intolerance, were consistent across all three generations indicating a transgenerational inheritance of metabolic dysfunction, particularly within insulin signaling pathways. Chronic HCD exposure in earlier generations likely impaired insulin sensitivity, triggering compensatory hyperinsulinemia. In the absence of dietary carbohydrate excess, the persistence of this phenotype in the F2 generation underscores the likelihood of inherited alterations in insulin responsiveness (Martins and Conde, 2022). Insulin resistance has been identified as a hallmark of metabolic syndrome and is closely associated with lipid dysregulation and excess fat accumulation (Guo, 2014; Veskovic et al., 2023). Furthermore, the interconnected nature of lipid and glucose metabolism suggests that the co-occurrence of dyslipidemia and hyperglycemia in F2 rats results from a coordinated disruption of metabolic control, initiated by ancestral diet and sustained through inherited gene expression changes (Ormazabal et al., 2018).

To better understand the molecular underpinnings of these phenotypes, gene expression analysis was conducted. A clear upregulation of glycolytic enzymes was observed in HCD-fed F0 and F1 generations, particularly glucokinase (GCK) and liver pyruvate kinase. These enzymes play central roles in glucose uptake and conversion into pyruvate, thereby supporting both energy production and lipogenic substrate supply (Agius, 2013; Schormann et al., 2019). Glucokinase is essential for glucose sensing in pancreatic β-cells and for hepatic glycogen synthesis, and a dysregulation of this enzyme has been associated with congenital hyperinsulinism and altered insulin secretion dynamics (Abu-Saad and Fraser, 2010; Abu Aqel et al., 2024; Matschinsky and Wilson, 2019). Pyruvate kinase, which catalyzes the final step of glycolysis, is also critical for energy metabolism and is regulated allosterically in response to cellular energy status (Martins and Conde, 2022; Lewandowski et al., 2020; Starling, 2021). Sustained upregulation of these enzymes in the F2 generation, despite dietary normalization, implies the inheritance of a metabolic state optimized for glycolytic flux and anabolic storage, which may contribute to insulin resistance and impaired lipid mobilization (Whitticar and Nunemaker, 2020).

Concurrently, the expression of phosphoenolpyruvate carboxykinase (PEPCK), a rate-limiting enzyme in gluconeogenesis, was significantly downregulated in HCD-fed generations and remained low in the F2 progeny. This aligns with previous research indicating that elevated insulin levels suppress hepatic gluconeogenesis by inhibiting PEPCK transcription and glucagon activity (Hatting et al., 2018; Leithner et al., 2015; Xu et al., 2018; Yu et al., 2021; Zhang et al., 2018). The suppression of PEPCK reduces the capacity for endogenous glucose production, particularly during fasting or glucose-limited states (Ferre and Foufelle, 2010; Kalra and Gupta, 2016; Petersen et al., 2017). This downregulation is mediated by insulin-sensitive transcriptional regulators, such as CREB, and reflects a metabolic adaptation to persistent carbohydrate availability (Dalle et al., 2011; He et al., 2020; Samuel and Shulman, 2016). The retention of this suppression in F2 animals suggests a heritable adjustment in gluconeogenic potential that may impair glucose regulation during energy stress.

Furthermore, glycogen synthase expression was elevated in the HCD-fed groups and persisted into the F2 generation. This enzyme plays a central role in hepatic and muscular glycogen storage by catalyzing the addition of glucose residues to glycogen chains (Adeva-Andany et al., 2016; Ferramosca et al., 2014). Insulin signaling promotes glycogen synthase activation through the PI3K-Akt pathway, which inhibits glycogen synthase kinase-3 (GSK-3) and supports increased glycogen deposition (Yang et al., 2023; Bouskila et al., 2008). Allosteric activation by glucose-6-phosphate further amplifies glycogen synthase activity, particularly under hyperglycemic conditions (Bruckdorfer KR and Yudkin, 1972; Pesta and Samuel, 2014). The continued upregulation of this gene in the F2 generation suggests that hepatic glycogen storage capacity has been epigenetically programmed, favoring glucose retention and possibly exacerbating insulin resistance under postprandial conditions (T., 2018). Lipogenic genes were also found to be consistently upregulated across generations. Elevated insulin levels and glucose concentrations activate SREBP-1c and ChREBP, which enhance the transcription of FAS and ACC, key enzymes involved in de novo fatty acid synthesis (Chiu et al., 2018; Iizuka, 2017; Lin et al., 2023). The accumulation of acetyl-CoA from glycolysis serves as a substrate for fatty acid synthesis, ultimately leading to triglyceride formation and fat storage (Menezes et al., 2013; Naismith DJ, 1974; PJ, 2002). This metabolic shift toward lipogenesis, particularly in the liver, contributes to lipid dysregulation and ectopic fat accumulation, and has been observed even in the absence of continued dietary carbohydrate excess (Saponaro et al., 2015). Conversely, a consistent downregulation of CPT1 and ACOX1 was observed. These enzymes are critical for the initiation of mitochondrial and peroxisomal fatty acid oxidation, respectively (Kashyap et al., 2023; McGarry and Brown, 1997). Reduced expression of these genes reflects a metabolic adaptation that prioritizes glucose as the primary energy source while suppressing lipid catabolism. This suppression is mediated in part by elevated insulin and malonyl-CoA levels, which inhibit fatty acid transport into mitochondria (McGarry et al., 1977; Randle et al., 1963; Schlaepfer and Joshi, 2020). Additionally, reduced PPAR-α activation diminishes the transcription of fatty acid oxidation genes, further limiting lipid utilization (Kersten et al., 1999; Mal et al., 2021; Zhao et al., 2020). This metabolic configuration promotes triglyceride accumulation and reduces energy flexibility, contributing to the development of insulin resistance and obesity (Hardie, 2012; Jeon et al., 2023; Nogueira-Ferreira et al.).

The study highlights a coordinated and inheritable reprogramming of metabolic pathways in response to high-carbohydrate feeding across generations. The persistent upregulation of glycolytic and lipogenic genes, along with the suppression of gluconeogenic and lipid oxidation enzymes, contributes to a phenotype characterized by increased fat accumulation, insulin resistance, and impaired metabolic flexibility. These traits remain evident in the F2 generation, which was never directly exposed to HCD, indicating the powerful influence of ancestral diet on offspring metabolism. This reinforces the significance of epigenetic inheritance in shaping disease susceptibility and underscore the need for preventive strategies that account for transgenerational nutritional effects. While our findings provide compelling evidence of transgenerational metabolic reprogramming following parental HCD exposure, a key limitation of this study is the inability to isolate the specific timing of dietary influence, such as exposures occurring exclusively during conception, gestation, or throughout life. It remains unclear whether the observed phenotypic and molecular alterations in F2 progeny are primarily driven by periconceptional nutritional environments, in utero programming, or cumulative lifelong exposure of the parental generation. Future studies designed to selectively manipulate maternal and paternal diets during discrete developmental windows, particularly during conception and pregnancy, will be critical to disentangle the timing and mechanisms underlying heritable metabolic programming. Such targeted approaches would refine our understanding of critical periods for intervention and enhance strategies to prevent the intergenerational transmission of metabolic dysfunction. Clinically, these findings have important implications for preventive medicine, emphasizing that dietary interventions in parents may be critical in mitigating transgenerational transmission of metabolic dysfunction. Targeted maternal nutritional strategies designed to normalize metabolic programming have demonstrated efficacy in reducing offspring susceptibility to obesity, insulin resistance, and related metabolic disorders (Fleming et al., 2018; Li et al., 2011). Such interventions could provide a practical approach to interrupting cycles of inherited metabolic disease, thus reducing the public health burden associated with metabolic syndrome and its complications.

## 6 Conclusion

The results of this study suggest that HCD-induced metabolic disruptions, such as insulin resistance and altered lipid profiles, persist in the F2 generation, through changes in gene expression of metabolic enzymes, even without direct HCD exposure, indicating potential epigenetic inheritance of metabolic dysfunction. Meanwhile, evidence of mechanisms of transgenerational epigenetic inheritance, including DNA methylation, histone modification, and Noncoding RNAs (microRNAs and long noncoding RNAs), was not examined. The study suggests, future studies that investigate transgenerational epigenetic mechanisms in F1 and F2 progeny not fed on the HCD of an exposed parent to strengthen the observed transgenerational changes in gene expression of metabolic enzymes and insulin resistance and altered lipid profiles revealed in this study. These findings may inform the development of early intervention strategies or preventive lifestyle recommendations in humans, particularly for populations with a family history of metabolic disorders.

## CRediT authorship contribution statement

**Joseph Chimezie:** Validation, Formal analysis, Writing – original draft, Writing – review & editing, Visualization. **Worship Odosa Agbonifo:** Validation, Formal analysis, Visualization, Investigation, Writing – original draft, Writing – review & editing, Visualization. **Hope Oluwabukola Francis:** Investigation, Formal analysis, Validation, Writing – original draft, Writing – review & editing, Visualization. **Mercy Oluwaseun Awoleye:** Investigation, Validation, Formal analysis, Writing – original draft, Writing – review & editing, Visualization. **Temitope Gabriel Adedeji:** Conceptualization, Methodology, Validation, Resources, Writing – original draft, Writing – review & editing, Supervision, Project administration.

## Ethical standards

The authors assert that all procedures contributing to this work comply with the ethical standards of the relevant national guides on the care and use of laboratory animals and have been approved by the institutional research ethics committee of the Federal University of Technology Akure, Nigeria with ethical approval number FUTA/ETH/21/02.

## Financial Support

This research received no specific grant from any funding agency, commercial or not-for-profit sectors.

## Declaration of competing interest

The authors declare that there are no known competing or conflicting financial interests or personal relationships that would have an influence on the work we have reported.

## Data Availability

Data will be made available on request.
